# ACE Enquiry in Primary care: A Qualitative Exploration of the Perspective of General Practitioners in Northern Ireland

**DOI:** 10.1007/s40653-024-00660-3

**Published:** 2024-09-28

**Authors:** Rafael Smyth, Dominic McSherry

**Affiliations:** 1 School of Psychology, Ulster University, Coleraine, Northern Ireland; 2 Reader in Psychology, Ulster University, Coleraine, Northern Ireland

**Keywords:** ACE, Trauma, Primary care, General practice

## Abstract

This study sought to identify gaps in the current literature base by exploring the perspectives of General Practitioners (GPs) in Northern Ireland in relation to the significance, relevance, and feasibility of conducting a comprehensive inquiry into Adverse Childhood Experiences (ACEs) with patients. Semi-structured, in-depth interviews were conducted with 10 qualified GPs using Zoom Videoconferencing technology. Interviews were audio recorded and transcribed verbatim. Qualitative data was analysed using Theoretical Thematic Analysis (Braun & Clarke Qualitative Research in Psychology 3(2):77-101, 2006). Analysis revealed nine key superordinate themes. These themes encompassed various aspects such as the role of a GP, trauma-informed training, the advantages and barriers associated with conducting an ACE assessment, and the impact of childhood adversity on subsequent physical and mental health. This study provides valuable primary care professional insights that contribute to the existing evidence base. It highlights the importance of recognising, discussing, and screening for ACEs in primary care settings. Furthermore, this study explores a range of practical adjustments that could support the implementation of routine ACE enquiry within the primary healthcare system in Northern Ireland.

## Introduction

### Prevalence of ACES in the United Kingdom

Adverse Childhood Experiences (ACEs) refer to severe and often chronic environmental and social events that occur during childhood, leading to profound physical and psychological harm to individuals. These experiences encompass various forms of abuse, such as physical, emotional, and sexual abuse, as well as neglect, parental separation, illicit drug use in the home, and parental incarceration (Kalmakis & Chandler, 2014). Despite the recognised limitations of specifying experiences only negatively, and almost singularly at the family level (Lacey & Minnis, 2019), Felitti’s (1998) seminal research did identify a strong and cumulative dose-response relationship between exposure to chronic stress and significant adversity during the formative years, which subsequently contributes to several risk factors associated with leading causes of death in adults, such as cancer, cardiovascular diseases, liver disease, and pulmonary illnesses.

Over the past two decades, there has been an increased scientific focus on conducting global studies to determine the prevalence of ACEs. These studies aim to enhance our understanding of how ACEs can serve as predictors of antisocial and health-harming behaviours during adolescence and adulthood. In England, it is estimated that 47% of adults have experienced at least one ACE, with 9% having experienced four or more ACEs (Bellis et al., 2014). Similarly, in Wales, it is estimated that 47% of the adult population has experienced at least one ACE, with 14% having endured four or more ACEs (Bellis et al., 2016). Despite Northern Ireland’s significant 30-year history of conflict, less is known about the prevalence of ACEs in the Northern Irish population. However, research conducted by McLafferty and colleagues (2015) concluded that 32% of the adult Northern Irish sample had experienced adversity during childhood. Furthermore, research conducted by the Northern Ireland Assembly (2017) has identified mental ill health as the leading cause of mortality and disability in the region, with rates of mental illness being higher in Northern Ireland than in any other jurisdiction in the UK, approximately 25% higher than in England (Betts & Thompson, 2017).

### Impact of ACES on Mental Health

A robust body of epidemiological and physiological evidence has been gathered which consistently links ACEs to mental ill-health and psychosocial consequences in adulthood. In a sample of over 9,000 individuals, Felliti and colleagues (1998) concluded that individuals who had experienced four or more ACEs were between four and 12 times more likely to suffer from depression, alcoholism, and drug abuse. Additionally, individuals with a cumulative ACE score of six or above, faced a significantly higher risk of shortening their lifespan by 20 years. These findings have been supported by McLafferty and colleagues (2015), who concluded that individuals in Northern Ireland who had experienced conflict and adverse childhood events were 15 times more likely to engage in suicidal behaviour and five times more likely to engage in self-harm.

ACEs have been shown to disrupt the development of key psychological functions, including adaptive emotive regulation processes and coping strategies (Sheffler et al., 2020), as well as the stress-response system (Merrick et al., 2017). Exposure to chronic stress and adversity in childhood has also been linked to behavioural risk factors and health-harming behaviours, such as alcohol and illicit drug dependency (Douglas et al., 2010) and sexual health risk behaviours (Felitti & Anda, 2010). Moreover, experiencing adversity in childhood has been specifically associated with incarceration in adulthood (Roos et al., 2016).

The economic impact of ACEs on public health and healthcare services is significant, with an estimated annual cost of 581 billion dollars in Europe (Bellis et al., 2019). Therefore, an enhanced understanding of the impact of ACEs and the implementation of evidence-based interventions to mitigate the harmful effects of ACEs could improve both the quality of life for millions of individuals and generate substantial cost savings.

### Impact of ACEs on Physical Health

The impact of ACEs on mental health functioning and the adoption of health-harming and risk-taking behaviours is one mechanism that links ACEs to poor health outcomes. However, emerging biomedical research has also identified a direct link between ACEs and neurological functioning, as well as the development of hormonal and immunological systems (Bellis et al., 2019). Concerningly, the presence of ACEs consistently serves as a risk factor for the diagnosis of chronic health conditions, including heart and lung disease, cancer, obesity, and diabetes (Huffhines et al., 2016).

Recent research has concluded that individuals who experienced parental separation or divorce, emotional abuse, or the death of a parent in childhood were at a significantly higher risk of being diagnosed with type 2 Diabetes Mellitus, compared to those who did not experience childhood adversity (Subramaniam et al., 2021). Additionally, the risk of being diagnosed with cancer before the age of 50 among women doubled for those who had two or more ACEs, compared to women with no ACEs (Kelly-Irving et al., 2013). A dose-response relationship between exposure to chronic stress and adversity in childhood, and a significantly higher risk of ischemic heart disease, has also been observed (Dong et al., 2004). Furthermore, a national health study in the United States found that exposure to four or more ACEs was associated with a significantly higher risk of cardiovascular disease (CVD), compared to individuals who had not been exposed to ACEs (Gilbert et al., 2015).

Children who have experienced ACEs are not only more likely to experience long-term physiological impairment, but may also undergo epigenetic changes in their bodies (Karlén et al., 2015). Research has demonstrated that newborn babies of mothers who had experienced four or more ACEs were five times more likely to experience poor physical and emotional health before the age of 18 months (Madigan et al., 2017). However, in this instance, it would be important to recognise that the impact of experiencing four or more ACEs would be likely to negatively impact parenting capacity, and hence may lead to the development of insecure attachment in children, that has a long-established association with poor longer-term emotional health (Bowlby, 1969).

The evidence base suggests that exposure to repeated adversity and toxic stress in childhood can lead to devastating long-term physical health consequences in adulthood. The impact of ACEs on the function of the Hypothalamic Pituitary Adrenal (HPA) axis has been recognized as an important pathomechanism to help explain this phenomenon (Clemens et al., 2020). There is a growing body of literature that suggests ACEs stimulate overactivation of the HPA axis, a key stress response system in the body. This overactivation, and subsequent increase in cortisol secretion, have been found to contribute to neural atrophy in the hippocampal area of the brain, immune system suppression (Doom & Gunnar, 2013), as well as somatic health conditions, including an increased risk for cancer, CVD, and metabolic diseases (Volden & Conzen, 2013).

### Role of General Practitioners in Primary Care

The literature extensively documents the strong and proportionate relationship between ACEs and the diagnosis of chronic physical and mental health conditions. The higher prevalence of physical and mental health difficulties among adults who have experienced childhood adversity highlights the significant need for healthcare providers to consider trauma-informed responses. Research has demonstrated that individuals rarely disclose their experiences of childhood adversity and trauma voluntarily. It is estimated that without direct questioning by professionals, it can take between nine and 16 years for a disclosure to occur (Schäfer et al., 2006). A study by Read and Fraser (1998) found that when asked directly, 82% of psychiatric inpatients disclosed experiencing trauma, compared to only 8% who volunteered this information without being asked.

Mental health poses a significant and unique demand on General Practice (GP), with figures suggesting that up to 90% of adults with mental health difficulties are solely supported in primary care (NHS England, 2016). Despite this, routine ACE enquiry is rarely the focus of primary care consultations (Green et al., 2011). GPs’ reluctance can be attributed to various factors, including fear of perceived consequences, lack of professional knowledge, lack of emotional support (Albaek et al., 2018), and insufficient training (Tink et al., 2017). Additionally, some researchers suggest that, in relation to GP contact with children, parents may view the interview process as too invasive, contributing to the reluctance. However, recent research found that parents were supportive of ACE screening as a means of accessing additional support, better understanding their children’s needs, raising awareness, and breaking the cycle of intergenerational trauma (Rariden et al., 2021). The literature suggests that having an enhanced understanding of an individual’s early life experiences of adversity and toxic stress may better enable GPs to provide the most appropriate care and support. Furthermore, this enhanced understanding may aid early intervention efforts to prevent the development of more complex mental and physical health complications and the reliance on prescribed drugs in the future (Hardcastle et al., 2020).

### Research Aim of the Current Study

In light of previous research, this study aims to explore the professional opinions of General Practitioners (GP) in relation to the importance, relevance and feasibility of screening for ACEs in primary care in Northern Ireland. It also seeks to consider their personal experience of utilising a trauma-informed approach and to consider potential barriers when screening for ACEs in practice.

## Method

### Participants and Sampling Procedures

A sample of ten qualified GPs, consisting of seven females and three males, voluntarily participated in the study. Convenience sampling was employed to recruit participants. The first author approached local GP Surgeries and created an online advertisement, inviting any interested GPs who met the inclusion criteria to participate. Participants were interviewed remotely using Zoom Videoconferencing technology. All participants were either currently practicing or had previously practiced within the Northern Irish Primary Health Care System. Prior to participating in the research, all participants were provided with a Participant Information Sheet (PIS) to ensure that they were able to make an informed decision and provide their consent. To protect the identity of participants, anonymous ID codes have been used. Participant characteristics are outlined in Table 1.

**Table 1:** Participant characteristics

Anonymous ID Code	Gender	Qualified Practice (years)
Participant 1	Female	6
Participant 2	Female	4
Participant 3	Female	15
Participant 4	Female	31
Participant 5	Female	29
Participant 6	Female	29
Participant 7	Male	5
Participant 8	Female	17
Participant 9	Male	6
Participant 10	Male	16

### Data Collection

Each participant took part in a comprehensive, in-depth interview lasting approximately thirty minutes, conducted using Zoom Videoconferencing. With the participants’ consent, the interviews were audio recorded and transcribed verbatim, with any identifying information removed. Comprehensive field notes were also taken during the interviews to enhance the reliability and validity of the findings (Low, 2013). The interview schedule was designed to align with the specific research aim and included questions such as “Do you believe that experiencing adversity in childhood has an impact on an individual’s mental health and well-being in adulthood? If so, in what ways?” and “In your opinion, what role does a GP have in initiating conversations with patients about ACEs?” These questions aimed to explore the professional opinions of GPs regarding the impact of childhood adversity on health outcomes and their views on whether it should be the responsibility of GPs to conduct a more in-depth examination of ACEs. The semi-structured interview schedule provided a framework for the interview process while allowing flexibility to explore relevant issues raised by the participants.

### Methodological Rationale

Guided by a review of the existing literature and with consideration of the study’s epistemology, theoretical Thematic Analysis (TA) was selected as an appropriate analytic method. In their seminal research, Braun and Clarke (2006) define TA as a rigorous and versatile method of organising, describing, analysing and translating qualitative data into key themes. Moreover, TA has been identified as a valuable method for systematically analysing health-related research, providing a unique opportunity for rich and detailed insight into the perspectives and expertise of health care professionals (Braun & Clarke, 2014).

### Analytic Process

Transcripts from the semi-structured interviews were analysed using Braun & Clarke’s (2006) Six Phases of Thematic Analysis (TA) Model. In keeping with the model, interview transcripts were read several times to facilitate researcher familiarity with the data corpus. The left- and right-hand side columns of the transcribed data was used to add additional annotations and analytic observations.

Following familiarisation, the data was reduced and organised in a meaningful and systematic way, using the qualitative data analysis software NVivo R1. Concise data labels were applied to the transcribed data highlighting important information relevant to the research topic. Following careful examination of the data codes, the data was analysed chronologically to identify recurrent themes which relate to the research topic. The first author thoroughly analysed, reviewed, and refined the initial themes to make sense of and ascertain connections between the emerging themes. The themes were analysed initially on a semantic level and then on a latent level to facilitate a deeper understanding of emerging ideas. A table of superordinate and sub themes was created (see Table 2). Finally, the qualitative analysis was written up as a narrative account, with conscious effort made to preserve the authenticity of participant accounts.

**Table 2:** Superordinate and organising themes identified from interviews

Superordinate Themes	Organising Themes
Unfamiliarity with term ‘trauma-informed practice’	
Training	Lack of training Psychiatry placement Autodidacticism
Role of a GP	Therapeutic relationship Educator Listening ear
Advantages of ACE enquiry	Reduction in healthcare seeking behaviour Increased GP time Financial incentive Best patient care Early intervention and prevention Raising awareness
Barriers to ACE enquiry	Time constraints Re-traumatisation Protection of own wellbeing Dearth of resources Jeopardising therapeutic relationship Panacea
Impact of ACEs on Physical Health	Somatisation Physical health conditions Indirect association
Impact of ACEs on Mental Health	Transgenerational trauma Substance abuse Maladaptive coping strategies Low self-esteem Relationship difficulties Emotion dysregulation Academic and social performance
Components of Mental Health Assessment	Psychosocial history Childhood Lifestyle factors Signposting
Lack of ACE enquiry	Not exploring ACEs during physical health assessment

### Ethical Considerations

Ethical Approval for the study was obtained from the *Author University* School of Psychology Staff and Students Ethics Filter Committee, in accordance with the British Psychological Society Code of Conduct and adhering to the General Data Protection Regulations (2018). Voluntary and informed consent was obtained from all participants before participation. All participants were informed that they could stop the interview at any point and withdraw their data up until two weeks post-interview with no risk of adverse consequences. To protect participant confidentiality, all identifiable information was removed from the transcribed interviews and participants were assigned an anonymous participant ID code. Audio recordings of the interviews and transcribed data have been stored securely with password encryption. Participant data will be held in a managed stored environment for 10 years in line with the University’s Research Data Management Policy.

## Results

Participant interviews clustered around nine superordinate themes: unfamiliarity with the term ‘trauma-informed practice’; training; role of a GP; advantages of ACE enquiry; barriers of ACE enquiry; impact of ACEs on physical health; impact of ACEs on mental health; components of mental health assessment; and lack of ACE enquiry (see Table 2). Each theme will subsequently be discussed and examples of quotations pertinent to those themes provided.

### Unfamiliarity with Term ‘trauma-informed Practice’

A recurrent theme among six out of ten participant interviews, was an unfamiliarity with the term ‘trauma-informed practice’. For some participants this was the first time they had heard the phrase and it was not something that they were familiar with in their professional practice (see Table 3).

**Table 3:** Unfamiliarity with term ‘trauma-informed practice’ with direct participant quotations

1. Unfamiliarity with term ‘trauma-informed practice’
Participant 1: “*Emm…well it is the first time that I have sort of heard that term from you.”*
Participant 2: “*Emm [pause] I suppose if I…to be honest it isn’t necessarily a term I would have heard of emm kind of previously.”*
Participant 3: “*Emm… I really don’t know actually.”*
Participant 4: “*Yes…not a lot probably. It’s not a term that I’ve heard before.”*
Participant 7: “*Yeah [pause] so it’s not a phrase that I’ve ever heard before so I’m just giving you my best impression of it.”*
Participant 8: “*I think that’s what I would take from that statement but it’s not something [pause] it’s not a term that I have*,* that I am overly familiar with.”*

### Training

This theme reflected participants’ views on the training that they had received during their professional careers which would enable them to initiate conversation about ACEs and engage in a more in-depth ACE enquiry with patients. Significantly, 90% of participants felt that they had received inadequate or no formal training on the topic matter during their academic study of medicine or specialist GP training (see Table 4). Subsequently, many of the GPs felt that they were ill-equipped and did not have the professional skills to conduct a trauma-informed ACE enquiry. Moreover, this theme was consistent among GPs regardless of how long they had been practicing professionally for, or how recently they had received their GP training.

**Table 4:** Training and organising themes with direct participant quotations

2. Training
a) Lack of training
Participant 1: “...*that niche discussing exploring childhood trauma I would say no I probably haven’t, you know had any formal training.”* *Participant 2: “...but bringing up something like that in any given consultation would be a completely different skill that as I say, I wouldn’t feel equipped.”* Participant 3: *“... but there has never been any formal education or anything like that, no.”* Participant 5: *“In a nutshell no and I can be perfectly honest and say [pause] it was only maybe, I am trying to think where I heard, it was only maybe a year ago that I heard… it must have been some training during Covid that I actually first ever came across the term ‘ACE’.”* Participant 6: *“Unfortunately, there has been a fortune put into, as you know in Northern Ireland there’s been a lot of money put in to developing emm [pause] sort of a programme raising awareness about ACEs. Emm [pause] but unfortunately, I don’t think primary care, have got it.”* Participant 10:” *I have received no training at all. I don’t…I don’t…I don’t know of any other GPs who have.”*
b) Psychiatry placement
Participant 1: *“I would say my psychiatry placement was invaluable in terms of making me feel comfortable dealing with risk for patients.”* Participant 4: *“So I happened to do a year psychiatry emm but a lot of GPs wouldn’t have done any...”* Participant 7: *“Emm [pause] we would touch on it when we do psychiatry as an undergraduate.”*
c) Autodidacticism*
Participant 5: *“I had a chance to really think about what that meant and do a little bit of reading myself. But other than that, there’s been [pause] I am not aware of any mandatory training or even any resource that you could go to look at.”* Participant 6: *“Basically I mean, whatever I know about ACEs it’s self-taught to be honest with you because I would do the training and I would seek out, seek out resources about ACEs when I’ve been doing the safeguarding because it’s such a big, massive part.”* Participant 7: *“So [pause] probably some of what I know, I would have learned myself as part of self-directed learning.”* Participant 10: *“I suppose feel quite strongly about it and have a decent understanding about it, is because for the last number of years I have been quite interested in, just in Psychology and lifestyle medicine. And also, through just exploring my own background and some of my, you know go on a journey with my stuff, emm so. But I most GPs…a lot of GPs probably haven’t even heard of ACEs, but I would say I…I don’t know of anyone who has had any actual training in it.”*

* Autodidacticism can be defined as self-directed learning or self-education

Some participants reflected that their Psychiatry placements had been helpful in providing them with an insight into the impact of mental ill health and developing skills in managing risk with patients. However, many felt that any knowledge and awareness they had of ACEs could be attributed to self-directed learning.

### Role of a GP

The role and responsibility of a GP in initiating conversation and facilitating exploration of ACEs during patient consultations was a key theme explored in the research. Some participants felt that GPs were ideally placed to conduct this sort of assessment, as often they are the first point of contact for patients (see Table 5). Additionally, some participants felt that due to the unique therapeutic relationship that GPs have with patients, and the advantage of knowing a patient holistically, GPs have a *“key role”* to play (Participant 7). Some participants reflected that the role of a GP should be as an educator, raising awareness and informing patients about the significant impact that experiencing adversity in childhood can have on later physical and mental health presentations. Additionally, the responsibility of a GP to be a ‘listening ear’ and source of support for their patients was a further opinion expressed.

**Table 5:** Role of a GP and organising themes with direct participant quotations

3. Role of a GP
a) Therapeutic relationship
Participant 1: *“It’s maybe reached a point where they want to open up and patients often feel more comfortable with a GP than a hospital doctor because the GP is maybe someone, they have known for years for their chest infection or their sore knee or things like that.”*
Participant 4: *“Emm, sort of over the period of my career, you would know the patients quite well, you would know their backgrounds and families. Emm [pause] so, we are at an advantage I suppose knowing the background story to some extent.”*
Participant 6: *“Yes it can be a real advantage because you know, you know the family and the wider family.”*
Participant 8: *“You know you’re the one that they trust.”*
b) Educator
Participant 6: “*Often educating them that they are behaving in this way, or they feel this way, or this has happened because of something that has happened in their childhood.”*
Participant 9: *“Yeah, [pause] so I mean I think sometimes it is helpful for the patient to understand where there is a parallel between a previous experience and what they are currently experiencing and sometimes that information can be helpful for patients.”*
Participant 10: *“You know emm, you know I think as a GP if we have an appreciation of the effects of trauma, we can maybe educate emm [pause] patients.”*
c) Listening ear
Participant 1: *“Just letting them talk and [pause] express what happened and be a listening ear and being sympathetic and allowing them time and not cutting them off.”*

### Advantages of ACE Enquiry

Many of the participants recognised the importance of conducting an in-depth ACE enquiry and identified a range of advantages of doing so, both for the patient and for the GP (see Table 6).

**Table 6:** Advantages of ACE enquiry and organising themes with direct participant quotations

4. Advantages of ACE Enquiry
a) Reduction in healthcare seeking behaviour
Participant 2: *“Emm and I suppose getting…if we screen for them perhaps a bit earlier, we may not get a situation where someone has already come to see me eh a number of times and actually it gets to the point where then they reveal this thing from the past.”*
Participant 8: “Emm, so I think maybe once you would establish that there is a pattern maybe.”
b) Increased GP time Participant 3: *“Well initially I would have said yes time, because mental health is saturating our workload.”*
c) Financial incentive
Participant 10: *“I mean we have been involved in various screening things over the years and there have always been financial incentives towards that...ehh so I think if there was some funding attached to being able to do the screening in general practice that would be, that would be very welcomed as well.”*
d) Best patient care Participant 5: *“Are there advantages? Yes, in the sense that it will give you a better idea you know of what your patient’s journey has been like to that stage. So yes, in terms of maybe understanding better what management might work for them.”*
Participant 8: *“I feel very strongly actually that if you can get to the core of who someone is, then you are much better placed to help them long-term.”*
*Participant 10: “I would say that [pause] it is probably one of the things that would have the biggest benefit to patients out of all of the things that we did... I think from the health, the physical and mental health perspective to me, that’s probably going to be the thing that is going to make the far biggest difference out of everything that modern medicine can do.”*
e) Early intervention and prevention
Participant 4: *“...and possibly [pause] emm an intervention at that stage could prevent long-term health issues.”*
f) Raising awareness
Participant 5: *“I think one of the things would be just to be aware of it in the consultation.”*
Participant 6: *“But…but I think if we even had more awareness of how important Adverse Childhood Experiences are to as you say, not just mental health presentations but also physical health as well.”*
Participant 9: *“So, emm I think this increased awareness is what primary care has the headspace for now rather than implementing a screening tool.”*

Participants highlighted the importance of GPs having an increased awareness of ACEs. With this additional knowledge, some participants felt that efforts could be made to intervene at an earlier stage and prevent the development of chronic, long-term health conditions. Additionally, some participants felt that conducting a more in-depth ACE enquiry with patients could help to get to the root of the difficulty and reduce healthcare seeking behaviour in the long-term, thus freeing up more GP time.

Participants also reflected that this type of assessment would enable GPs to have a more comprehensive understanding of their patients’ presenting difficulties and ensure that they receive the best care by signposting them to the appropriate supports.

### Barriers to ACE Enquiry

While participants could appreciate the advantages of conducting an in-depth ACE enquiry, they also identified a range of barriers which would make the implementation of this challenging in practice (see Table 7). One of the main barriers, highlighted by all participants, was the constraint of time. Participants felt that the allocated 10-minute consultation time with patients did not allow for a more in-depth exploration of childhood adversity and trauma and GPs often felt *“constrained to just deal with the issues in front of you”* (Participant 5).

**Table 7:** Barriers to ACE enquiry and organising themes with direct participant quotations

5. Barriers to ACE Enquiry
a) Time constraints Participant 2: “*Emm [pause] I suppose my only concern about that would be how to do it in a you know ten-minute consultation about someone’s diabetes to say…to bring that up it, I wouldn’t know how to kind of skilfully bring that in.”*
Participant 3: *“Emm and I think I would love to have the luxury of spending a lot of time…but unfortunately, we are not [laugh] counsellors.”*
Participant 4: “I *suppose it’s the old chestnut of the time that we have. We have to deal with all our populations’ health needs and try and prioritise them.”*
Participant 5: *“Yeah no absolutely and I mean [pause] time is the biggest barrier to all sorts of things that we should be doing.”*
Participant 7: *“I mean we’ve ten minutes so it’s not a Psychiatrist’s time that they have to go through childhood and all of that [pause].”*
b) Re-traumatisation Participant 2: *“Emm, but again my worry would be delving into something and then the patient leaving with these open wounds because of past trauma and then not having somewhere immediately to try and deal with that. I feel like I could do harm actually to people.”*
Participant 5: *“Emm [pause] and I think the other thing as well to be perfectly honest is, you’re potentially opening a can of worms in terms of that trauma.”*
Participant 6: *“You’re opening up a Pandora’s box often for people when you start talking about it and unless you have somewhere to refer them to, you might as well not even talk about it.”*
c) Protection of own wellbeing
Participant 6: *“I mean sometimes the barrier also, I mean I always use this as a barrier when I’m talking is, well, I suppose the emotion, your own…your own emotional emm you know.”*
d) Dearth of resources
Participant 1: “The mental health services you know, are running on fumes you know as well and then we don’t have enough Psychologists.”
Participant 5: *“You know we’re resource poor at the minute you know we don’t have enough GPs, we don’t have enough other staff.”*
Participant 9: *“Emm, and effectively we have a very high workload, high demand and not a huge amount of resources to match that so I think there is always a tension between what we could do you know in terms of ideal verses what we can do in terms of practicalities.”*
e) Jeopardising therapeutic relationship
Participant 7: “*And certainly when you refer somebody into secondary care mental health services, if you mention that, they will ask you have you reported this to the police or have you told them to report this to the police and that, like that really imperils your therapeutic relationship as a doctor if it’s maybe someone who has opened up to you about something that they haven’t talked about before.”*
Participant 8: *“You know, so you don’t want to be dismissive either because it completely negates their trust in you, in that relationship if they’ve shared something with you.”*
f) Panacea
Participant 1: *“But then at that point that is when GPs instantly hit the wall because you’ve got patients in crisis who are unwell, wanting something understandably to fix them.”*
Participant 8: *“I mean there are people who you know pretty much as you begin to talk to them know that they are not going to engage, and it is pretty much up to you as a doctor to fix them.”*

The GPs interviewed also expressed a reluctance to explore history of ACEs out of fear of re-traumatising the individual and potentially jeopardising the therapeutic relationship that they had established. Participants reported feeling under pressure at times to find a panacea for their patients in crisis, who *“wanted something*,* understandably*,* to fix them”* (Participant 1).

A further barrier highlighted by participants was a dearth of resources in primary care. This included a lack of full-time working GPs, saturated caseloads, lack of psychological skills or training and extensive waiting lists for statutory mental health services.

### Impact of ACEs on Physical Health

There was a general consensus among participants that experiencing adversity and toxic stress in childhood adversely impacts an individual’s physical health presentation in adulthood (see Table 8). A recurrent organising theme raised during discussion was ‘somatisation’. This referred to the manifestation of a patient’s psychological trauma as physical health complaints in the body. Recurrent examples of these physical health complaints offered by participants included: chronic pain, fibromyalgia, chronic fatigue, rashes, and gastrointestinal disorders.

**Table 8:** Impact of ACEs on physical health and organising themes with direct participant quotations

6. Impact of ACES on Physical Health
a) Somatisation Participant 1: *“Your mental health could be a huge part; this could be your body’s physical representation of how you are doing mentally.”*
Participant 8: “*I don’t know maybe psychosomatic symptoms that a child or a young person or an adult or an older adult might present with.”*
Participant 9: *“Yeah well, I think it is hard to totally separate, isn’t it? But we definitely see increased somatisation.”*
b) Physical Health Conditions
Participant 3: *“I mean a lot of patients with mental health problems would present with probably the big things: tiredness, bowel disorders and rashes and sometimes you break it all down or memory loss actually another one. When you break it all down, depression or some form of trauma is the underlying aspect of it you know.”*
Participant 6: *“So things like fibromyalgia, chronic pain, certain neurological conditions emm irritable bowel syndrome, gynaecological conditions as well particularly chronic pelvic pain but it’s often things like even, obviously typically emm sexual dysfunction can be associated with abuse as well definitely we know that.”*
Participant 8: “*You know the irritable bowel, the headaches, the anxiety you know not necessarily just the psychological, but you know people with physical symptoms that might relate to that. Ulcers, tummy ulcers you know other things like that.”*
c) Indirect Association Participant 7: *“Yeah very much so [sigh] as well. Emm, ehh…probably indirectly.”*
Participant 9: *“In terms of physical illness, emm I am not sure whether ACEs are associated directly, [pause] although they definitely are associated with other forms of social deprivation indexes.”*

While participants broadly agreed that there was an association between ACEs and physical health, some participants felt that this was an indirect association that was significantly influenced by an individual’s decision to engage in risk taking and health harming behaviours.

### Impact of ACEs on Mental Health

All of the GPs interviewed agreed that experiencing ACEs could have a detrimental impact on an individual’s mental health and emotional wellbeing in adulthood. Experiencing low self-esteem, relationship difficulties, emotion regulation difficulties and engaging in maladaptive coping strategies were some of the ways that participants believed this manifestation of psychological trauma could be demonstrated (see Table 9). Participants reflected that working longitudinally with families in the community, provided them with an insight into the harmful effect of transgenerational trauma within a family system. They alluded to the patterns of behaviour and “*coping mechanisms or the lack of them that are transmitted from one generation to another*” (Participant 4).

**Table 9:** Impact of ACEs on mental health and organising themes with direct participant quotations

7. Impact of ACEs on Mental Health
a) Transgenerational trauma
Participant 3: *“Oh absolutely, we see it now through the generations, so we could see patients coming in with babies and we’ll almost predict that we are going to see these children [pause] in the next number of years, with the same [pause]. So, the patterns are there, and they continue.”*
Participant 4: *“Yeah absolutely, how coping mechanisms or the lack of them are transmitted from one generation to another.”*
Participant 5: *“I hate to call it, history repeating itself but sometimes you know if you start to ask questions about certain circumstances or certain behaviours that patients have, it can start to come up that it is experiences that they have lived through.”*
b) Substance abuse
Participant 4: *“Emm [pause] and, you know substance abuse as well I suppose is a mental health reflection.”*
Participant 7: *“Also associated often with polysubstance abuse, alcohol abuse or excess, risk-taking behaviours or interesting behaviours as well.”*
Participant 9: *“One of the main ones being substance misuse as a coping strategy to deal with psychological trauma.”*
c) Maladaptive coping strategies
Participant 1: *“Option of convenience, you know so fast foods, alcohol to numb sort of the thoughts and feelings.”*
Participant 6: *“It even effects things like, emm [pause] they’re more likely to be smokers, they’re more likely to have poor eating habits so therefore more likely to be obese, you know.”*
Participant 10: *“You know, as a result of you know, not looking after themselves properly, engaging in risk-taking behaviours etcetera.”*
d) Low self-esteem
Participant 1: “*If it has been sexual trauma a lot of them have very low self-worth after that and they don’t look after themselves.”*
Participant 9: *“Emm low self-worth also, and how this can influence how individuals deal with further trauma strains also.”*
Participant 10: *“Emm, I think if it became apparent that this person was struggling from, you know some kind of chronic stress or you know, they did have you know unhealthy behaviours which could be linked to low self-esteem or anxiety or something like that.”*
e) Relationship difficulties
Participant 3: *“So definitely to me, if you have had some sort of abuse or trauma in the past that hasn’t been dealt with it’s really just sort of stored up and it’s going to come out eventually so that can affect your interactions with your partner, so you know developing relationships.”*
f) Emotion dysregulation
Participant 9: *“Yeah absolutely [pause] it really emphasises how important the formative years are and shows how ACEs can perpetuate emotional instability.”*

### Components of Mental Health Assessment

This theme encompassed the aspects of assessment that GPs would routinely explore when an individual presented with a mental health difficulty. This assessment involved an in-depth biopsychosocial exploration of the individual’s psychosocial history, childhood experiences, lifestyle factors and current medication use, considering how these factors may be influencing the patient’s current mental state (see Table 10). Following this thorough assessment, participants alluded to the importance of *“signposting to appropriate resources”* (Participant 7) and making a referral to the mental health team who could best support the patient. As within previous themes, frustrations regarding the extensive waiting lists for statutory services and the lack of funding for mental health care were also expressed.

**Table 10:** Components of mental health assessment and organising themes with direct participant quotations

8. Components of Mental Health Assessment
a) Psychosocial history
Participant 5: *“So, I try to [pause] take a good social history and try and maybe delve into that with folk, especially if I think there are emm parts of the person’s history that would lead me to think that there might be something behind that.”*
Participant 7: *“And then depending on what the condition is, you will think a wee bit about psychological and social things.”*
Participant 10: *“I would always try to explore whether they may be currently in a situation in which they are exposed to trauma, or whether they maybe have been exposed to trauma at some point in the past.”*
b) Childhood
Participant 2: *“I mean if people talk about having had something ongoing from when they were a child, low mood, anxiety, I would ask a generalised question like you know how was your…how was your childhood and are things ok at home?”*
Participant 5: *“It is just about being aware that there might be things in their experience or even back in their childhood.”*
Participant 6: *“But I think the key to it is [pause] asking the difficult questions, the direct, sometimes you have to ask direct questions because pretty often adults have forgotten what their childhood…the impact that their childhood was having.”*
c) Lifestyle factors
Participant 1: *“Emm… [pause] it really is [pause] exploring lifestyle.”*
Participant 2: *“Emm I suppose it would involve their lifestyle in terms of their exercise and their…so a lot of the time mobility comes into that actually, you know people who can’t exercise because of poor mobility, their diet emm [pause].”*
Participant 5: *“So, if they’re at risk of, it centres a lot around their lifestyle emm [pause] so you are asking them sort of about their dietary history, exercise what they do or don’t do. You’re asking them about their smoking history, you’re asking them about you know emm any other sort of drug history or alcohol.”*
d) Signposting
Participant 1: *“Are we referring and if we are referring who are we referring to? Is it just to counselling or CBT or is it to the mental health team for someone specific emm like Psychology or help with medication? Or risk management? Or am I prescribing?”*
Participant 7: *“So, then that would be when we explore those things a little bit, usually then we need to signpost them to appropriate resources.”*

### Lack of ACE Enquiry

While most participants agreed that there was a relationship between experiencing ACEs and subsequent poor physical health in adulthood, none of the participants interviewed stated that they would explore the history of ACEs during an assessment with an individual presenting with a physical health condition, such as pre-diabetes (see Table 11). Some participants reflected that this was not something that would be on their radar when an individual is not presenting with a mental health difficulty. Other participants reflected that this is something which might be explored at a later date, if the patient was to offer this information.

**Table 11:** Lack of ACE Enquiry and organising themes with direct participant quotations

9. Lack of ACE Enquiry
a) Not exploring ACEs during physical health assessment Participant 2: *“No to be honest with you it wouldn’t have ever been something that I would have thought of.”*
Participant 3: *“But we would never formally ask about childhood traumas or anything like that [pause] no.”*
Participant 5: *“To be perfectly honest with you*,* if I had either of those two presentations in front of me*,* I would not be thinking of adverse childhood emm episodes*,* [pause] I wouldn’t to be clear.”*
Participant 6: *“Emm to be honest with you no. Not really now*,* to be honest with you. Emm unless it came up.”*
Participant 9: *“But in terms of ACEs or other psychological factors they wouldn’t necessarily be involved during my assessment of a client who was pre-diabetic.”*

## Discussion

The detrimental impact of childhood adversity on an individual’s mental health has been extensively documented in the literature. Recent data has also revealed a strong and proportionate relationship between childhood adversity, toxic stress, and the diagnosis of chronic health conditions such as Type 2 Diabetes, Heart Disease, Respiratory Disease, and Cancer (Larkin et al., 2014). The higher prevalence of physical and mental health difficulties among adults who have experienced childhood adversity, or at least the range of adversities specified in the ACE questionnaire, highlights the significant need for healthcare providers to respond in a trauma-informed way. However, routine enquiry of ACEs has not yet been incorporated into the primary healthcare system in Northern Ireland. Therefore, this exploratory study aimed to gain a greater understanding of GPs’ awareness and knowledge of the impact of ACEs, as well as their professional opinions on the relevance and feasibility of incorporating routine ACE enquiry into primary healthcare assessments in Northern Ireland.

The study identified nine key superordinate themes based on the results. Firstly, six out of ten participants reported being unfamiliar with the term ‘trauma-informed practice’. This finding was surprising given the strong impetus behind creating a trauma-informed workforce in Northern Ireland and recent significant financial investment in the Early Intervention Transformation Programme (EITP) Trauma Informed Practice Project in 2018 (Housing Executive NI, 2019). However, this finding is consistent with previous research conducted by Szilagyi et al. (2016) which found that 76% of Paediatricians included in their sample were not familiar with the original ACE study.

Participants demonstrated good awareness of the detrimental impact of childhood adversity and toxic stress on mental health and emotional wellbeing. Their professional knowledge was evident in their biopsychosocial approach to mental health assessments. GPs acknowledged the significance of considering psychosocial history, lifestyle factors, and adverse childhood experiences when assessing patients who presented with mental health difficulties. In contrast, none of the GPs interviewed mentioned that they would explicitly enquire about ACEs if a patient presented with a physical health complaint, such as pre-diabetes. Their knowledge of the association between ACEs and physical health was less common, with many GPs viewing it as an indirect association resulting from engagement in risk-taking and health-harming behaviours. These findings are consistent with existing literature that established physicians’ knowledge of the association between ACEs and mental health, while knowledge of the association with physical health was significantly less common (Maunder et al., 2020). Another study found that only 34% of Paediatricians agreed that prolonged exposure to toxic stress in childhood could result in DNA modification (Szilagyi et al., 2016).

A key theme discussed during the interviews was the role and responsibility of a GP in facilitating the exploration of ACEs during consultations with their patients. There was a general consensus among participants that GPs were ideally positioned to conduct this type of assessment. Participants reflected on the unique advantage and privileged position they held in knowing their patients throughout their lives and gaining deep insight into their family backgrounds. The trusting relationship established between patients and GPs was also reflected in contemporary ACE literature. In a sample of patients interviewed by Goldstein et al. (2017), most reported feeling comfortable when directly questioned by their GP about personal experiences of ACEs, and most felt at ease with the inclusion of these results in their medical records.

Moreover, a significant finding of the current study was the collective feeling of inadequate training among participants. Nine out of the 10 participants did not feel they had received professional training on how to initiate conversations about ACEs with patients. This finding corresponds with a recent large-scale global systematic review that identified inadequate training and lack of formal knowledge as the most frequently cited barriers to ACE enquiry among primary care practitioners (Ashe et al., 2022).

Participants in the current study also expressed concerns about potentially causing harm and re-traumatizing their patients. These concerns align with the earlier work of Green et al. (2011), where GPs felt reluctant to explore topics they had not been prepared for out of fear of causing further harm and invading patients’ privacy. However, contrary evidence has shown that a significant majority of patients (79%) felt comfortable being asked about their experiences of ACEs, and 86% felt comfortable being screened for ACEs by their GP, regardless of whether or not they had experienced adversity in childhood (Goldstein et al., 2017).

Another key barrier raised by GPs during the semi-structured interviews was the constraint of a 10-minute consultation slot with patients. A scoping review published in 2023 acknowledged clinic workflow as an important variable that could reduce the feasibility of ACE enquiry in routine medical appointments. However, the review concluded that incorporating this enquiry was not time-intensive or resource-heavy (Mishra et al., 2023). One study reported that conversations about ACEs with patients lasted between 3 and 5 min on average (Gillespie & Folger, 2017).

Additionally, for individuals with a high ACE score, the average visit time increased by five minutes or fewer, and no visits increased by more than 15 min (Glowa et al., 2016). Moreover, participants reflected on the potential impact of exploring ACEs on their own mental health and emotional wellbeing, echoing existing literature where primary care health providers acknowledged that screening for ACEs was personally distressing (Pearce et al., 2019).

In the current study, GPs reported that one of the key benefits of introducing a more in-depth ACE enquiry would be to raise awareness and educate patients about the impact of ACEs on physical and mental health. This theme aligns with the findings of Campbell et al. (2020), where many GPs recognized that initiating ACE conversations allowed them to “plant a seed” for further discussion. Physicians also recognized the importance of using ACE discussions as an educational tool to establish the intrinsic link between physical and mental health (Kia-Keating et al., 2019). Additionally, some participants reflected that introducing a more in-depth ACE enquiry into routine patient assessments may help reduce healthcare-seeking behaviour in the long term. This hypothesis was tested by Larkin et al. (2014), who observed a 35% reduction in visits to GPs and an 11% reduction in Emergency Department visits when patients were asked about their experiences of ACEs during a standard medical assessment.

### Limitations of the Current Study

There were a number of potential limitations in the study that should be considered when evaluating the findings. Firstly, the study was qualitative in nature, and as such, could not provide findings that would be generalisable to the wider GP population in Northern Ireland. Further research should be conducted to develop a survey that can be used with a representative sample of GPs across Northern Ireland, with the current study helpfully providing a range of topics for larger-scale quantitative consideration. Furthermore, there was a significant gender imbalance within the sample, with more female (*n* = 7) than male (*n* = 3) participants, which made it difficult to explore the data on the basis of gender in any meaningful way. Again, a larger-scale survey of GP opinions would help ensure that gender could be accounted for, with greater effort made to ensure an increased level of participation by male GPs.

Additionally, convenience sampling was used to recruit participants. Research has indicated that such sampling procedures may increase the risk of self-selection bias and potentially compromised the internal validity of the study (Tripepi et al., 2010). Participants who chose to participate may have stronger views or a particular interest in the topic, leading to a potential bias in the findings. Future qualitative research should consider employing a more robust sampling strategy to enhance the representativeness of the sample. Also, to enhance the quality and rigor of the study, respondent validation could have been included to ensure the accuracy of the researcher’s interpretation of the findings and to confirm that they truly reflect the participants’ views (Mays & Pope, 2000).

It is also important to acknowledge the ongoing debate surrounding the clinical efficacy of the ACEs questionnaire. Concerns have been raised that the ACE tool is too negatively orientated, and as such, is not capable of measuring protective factors in the lives of children, which can influence physical and mental health outcomes for children exposed to early adversity (Lacey & Minnis, 2019). Furthermore, the ACE tool is singularly located at the family-level, thus ignoring important community and societal level adversities, such as poverty (Marmot et al., 2020) and racism (Bernard et al., 2022).

Additionally, the psychometric properties of the measure have been criticized (Racine et al., 2020) and concerns have been raised regarding the potential mandatory reporting to child protection services and the potential stigma associated with ACE screening, which may lead to disengagement from services among families (Campbell, 2020).

These critiques should be taken into account when considering the implications and limitations of the study’s findings. It may also be worth noting that any potential specific deficits in the ACEs questionnaire itself should not diminish the clear evidence of the general negative impact of adverse childhood experience.

### Reflexive Perspective from the Authors

For the first author, it has been a privilege to engage with GPs on such a critical, yet under-researched topic. The interest in the research area of Adverse Childhood Experiences (ACEs) and health outcomes stems from a personal family experience involving a chronic diagnosis that could not be fully explained by physical health markers alone. This research has emphasised the profound importance of integrating ACE consideration and fostering open discussions about trauma during primary care consultations. It has highlighted the importance for frontline healthcare professionals to be equipped with the skills and resources necessary to provide holistic assessments and comprehensive follow-up care. It is hoped that these findings will have real-world implications, contributing to the improvement and transformation of primary care services in Northern Ireland. Heartfelt gratitude is extended to all of the participants for their valuable time and contributions, without which this research would not have been possible.

For the second author, interest in this area of work is reflective of a growing professional and personal appreciation of the need to widen understanding of psychological trauma, and its potential aftermath, across all echelons of society, particularly those areas of professional working where there is a direct interface with those who are likely to be suffering from the legacy of early childhood trauma. Being trauma-informed should help those who work directly with children and adults who have experienced trauma respond to their needs in a more timely and sensitive way.

### Implications for Practice

Despite the potential limitations, the present study highlights important factors that should be considered in the implementation of best clinical practice guidelines. The lack of consultation time with patients emerged as a key barrier, making ACE enquiry impractical for GPs. Research has shown that tailoring the length of GP consultations to meet individual patient needs improves comprehensive biopsychosocial assessment and the provision of preventative care (Brooks et al., 2018).

Additionally, participants strongly expressed the need for formal training for GPs on how to initiate conversations about ACEs with patients and conduct ACE screening in a sensitive and meaningful manner. The REACh (Routine Enquiry about Adversity in Childhood) screening tool developed by the Public Health Department in Blackburn with Darwin Council is an example of an approach that has shown positive results. A qualitative evaluation of this model demonstrated that professionals who were adequately trained and supported in using the screening tool, felt confident in facilitating discussions around ACEs and believed it was valuable, leading to improved patient outcomes. Concerns about increased service demand were unfounded, as individuals were able to access appropriate support (McGee et al., 2015).

Finally, the issues raised in this study regarding the challenges faced by General Practitioners in addressing the potential ACE history of their patients highlights a particular regional concern, given that rates of mental illness are higher in Northern Ireland than in any other jurisdiction in the UK, and approximately 25% higher than in England (Betts & Thompson, 2017). The existence of this elevated level of mental health difficulty in Northern Ireland is something that should be highlighted in any future training for trainee and practicing General Practitioners working or planning to practice in Northern Ireland.

## Conclusion

This study aimed to investigate the professional perspectives of General Practitioners concerning the significance, pertinence, and feasibility of ACEs (Adverse Childhood Experiences) screening within the context of primary care in Northern Ireland. The unique opportunity to conduct in-depth interviews with ten experienced GPs in Northern Ireland, contributes substantial professional insights to the existing body of evidence, underlining the imperative to acknowledge, engage in discussions about, and undertake ACEs screening within primary care settings. Furthermore, it offers a comprehensive assessment of the various impediments, including resource limitations, insufficient formal training, and time constraints, which render ACE inquiry a more challenging endeavour. Consistently, research has demonstrated that relying solely on individuals to spontaneously disclose their histories of trauma or adversity is an ineffective approach, as many years may elapse before such information is voluntarily shared (Read et al., 2006). Hence, the findings of this present study should be taken into careful consideration when determining how to effectively implement ACEs inquiry within the primary healthcare system in Northern Ireland.

## Data Availability

The participants of this study did not give written consent for their data to be shared publicly, so due to the sensitive nature of the research, supporting data is not available.
